# Tracing and tracking filamentous structures across scales: A systematic review

**DOI:** 10.1016/j.csbj.2022.12.023

**Published:** 2022-12-16

**Authors:** Isabella Østerlund, Staffan Persson, Zoran Nikoloski

**Affiliations:** a Department of Plant and Environmental Sciences, University of Copenhagen, 1871 Frederiksberg, Denmark,; b Bioinformatics, Institute of Biochemistry and Biology, University of Potsdam, 14476 Potsdam, Germany; c Systems Biology and Mathematical Modeling, Max Planck Institute of Molecular Plant Physiology, 14476 Potsdam, Germany

**Keywords:** Cytoskeleton, Filamentous Structures, Image processing, Tracing, Tracking, Deep learning, Filamentous structure, FS, Microtubule, MT, Actin filamentous structure, AFS, Machine learning, ML, Convolutional Neural Network, CNN

## Abstract

Filamentous structures are ubiquitous in nature, are studied in diverse scientific fields, and span vastly different spatial scales. Filamentous structures in biological systems fulfill different functions and often form dynamic networks that respond to perturbations. Therefore, characterizing the properties of filamentous structures and the networks they form is important to gain better understanding of systems level functions and dynamics. Filamentous structures are captured by various imaging technologies, and analysis of the resulting imaging data addresses two problems: (i) identification (tracing) of filamentous structures in a single snapshot and (ii) characterizing the dynamics (*i.e*., tracking) of filamentous structures over time. Therefore, considerable research efforts have been made in developing automated methods for tracing and tracking of filamentous structures. Here, we provide a systematic review in which we present, categorize, and discuss the state-of-the-art methods for tracing and tracking of filamentous structures in sparse and dense networks. We highlight the mathematical approaches, assumptions, and constraints particular for each method, allowing us to pinpoint outstanding challenges and offer perspectives for future research aimed at gaining better understanding of filamentous structures in biological systems.

## Graphical Abstract

**Figure:**
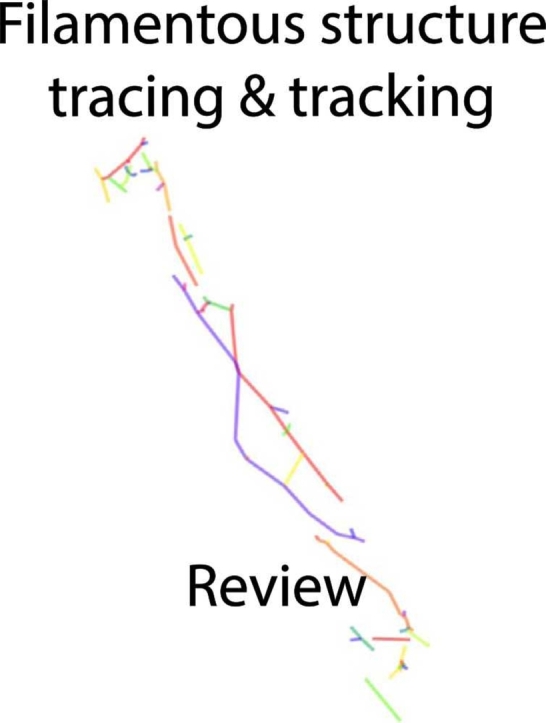

## 1 Introduction

Filamentous structures (FSs) are thread-like objects that are ubiquitous and cover different spatial scales: from the cytoskeleton inside a cell [1] and venation patterns of leaves [2] to vascular systems in diverse organisms [3] as well as solar filaments [4] and galaxies [5]. FSs perform multiple diverse functions in biological systems. For instance, the cytoskeleton aids in maintaining cell shape, cell organization and in carrying out essential cellular functions like division and movement [1], [6]. The vascular system supports flow of bodily fluids, while the retinal blood vessels carry oxygen and nutrients to the eye cells [7]. Therefore, accurate identification of FSs along with characterization of their properties represent important research problems to understand the function of FSs with the potential of diverse applications.

To this end, robust tools for FS analysis can provide quantitative characterization of their structures and dynamics. This is typically analyzed by human annotation [8], [9], [10], [11], which may be feasible for *in vitro* systems where there is little to no overlap between FSs. However, manual tracing of FSs becomes unfeasible for systems where FSs overlap, rendering their identification challenging even for experts. In addition, manual identification of FSs by tracing may also introduce bias, depending on the level of expertize of the users.

Another, more robust way to analyze FSs relies on the usage of algorithms along with their implementations in automated tools and pipelines. Such automated tools use images of FSs as input, and often require image preprocessing to remove noise and to enhance FSs; this is achieved by application of conventional filters *e.g.* Gaussian filter followed by line enhancement filters [12], [13], [14]. Subsequent steps usually include: (1) segmentation of images, (2) tracing of individual FSs, and, in the case where time-resolved images are provided as input, (3) tracking of FSs. Each of these steps represent particular computational problems that entail the design and usage of different algorithms.

**Fig. 1:**
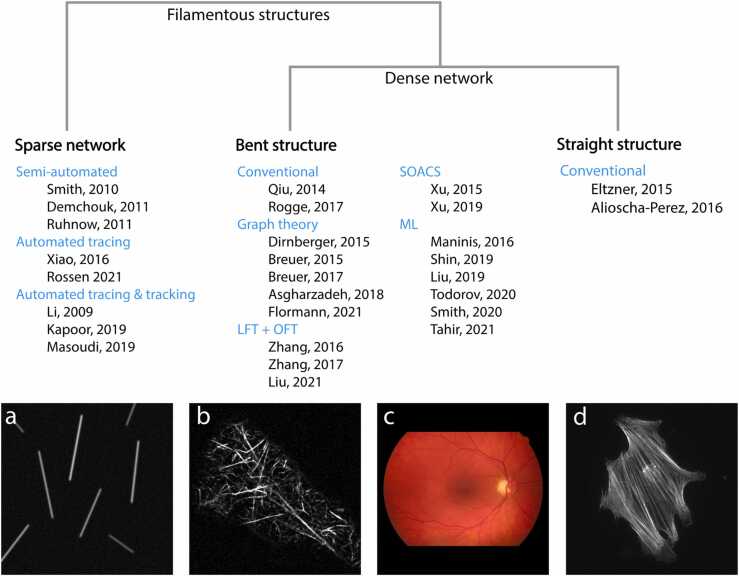
**Classification of approaches to identify and track filamentous structures.** The existing methods for tracing and tracking of FSs are grouped based on the type of input they receive, *i.e*. sparse or dense networks. The approaches dealing with dense networks are grouped based on whether they consider straight or bent FSs. The resulting three subgroups are further refined based on properties of individual approaches. Conventional refers to approaches that rely on standard image processing techniques. (a) Simulated image of single microtubule filaments in vitro. (b) Image of actin cytoskeleton in pavement leaf cell from Arabidopsis thaliana. (c) Eye-fundus photograph of the human eye from the HRF database [73]. (d) Image of actin stress fibers from Eltzner et al. [33].

FSs form networks that lend themselves as a theoretical framework in which the problems of tracing and tracking of FSs can be readily studied. In this regard, the approaches for automated tracing of FSs can be further categorized based on the FS network density, namely, sparse and dense networks (Fig. 1). Sparse networks are composed of FSs that rarely or never cross, resulting in networks with many sparse pieces (referred to as connected components). For instance, such sparse networks arise in *in vitro* diluted experiments with cytoskeletal components (see Fig. 1a), venation, root, and river network systems, in which every connected component is tree-like (*i.e*., has no or very few cycles). By contrast, dense networks typically include FSs that cross and overlap, creating loops on themselves, and result in a large number of cycles as representative network structures. Such dense networks are typical for the cytoskeleton inside cells and retinal vasculature (Fig. 1b,c).

Aside from manual annotation, tracing and tracking of FSs can be performed in fully or semi-automated ways, which affect the findings from the analyses. Semi-automated approaches require input from the user; by contrast, fully automated analyses operate without user’s intervention and can control any bias due to user’s input. Irrespective of the type of automation, tracing and tracking usually require usage of parameters to fine-tune the performance of particular algorithmic steps. While a large number of parameters allows for flexibility, it may bias the findings or lead to artifacts if parameter selection is not adequate. This can be avoided, to a certain extent, by learning (*i.e*., selection) of parameter values based on the input data used in tracing and tracking of FSs.

**Fig. 2:**
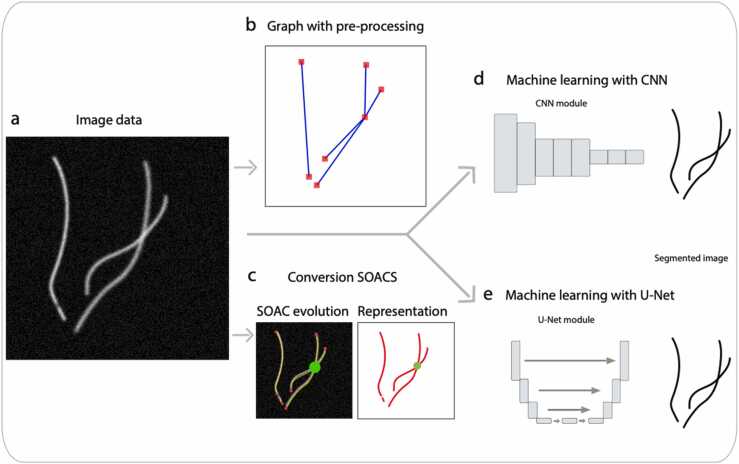
**Schematic explanation of approaches for tracing of filaments**. (a) In silico data with noise representing filament-like imaging data. (b) Imaging data translated to planar graph drawn with spatial coordinates, after preprocessing to enhance filament-like structures. (c) Stretching open active contours (SOACS) mathematical model fitted directly on imaging data. Each fitted open active contour function is illustrated as a red line, crossing points are illustrated with a green circle. Tracing and segmenting filament-like structures based on (d) Deep learning machine learning approach using a Convolutional Neural network module which takes the input image and assign importance to various aspects of the image. (e) U-Net approach where the U-shape represents first original image input, followed by a contracting path where spatial information is reduced while feature information is increased, and lastly followed by the expansive path where image features and spatial information is combined, resulting in a mask the size of the original image.

Individual FSs can be readily identified and tracked in sparse networks, representing image-data, using well-established algorithms for particle detection and localization problems. This is achieved by subtracting images from each other and analyzing the subtracted signal only. However, analogous approaches are not applicable for dense networks, where FSs may be bundled and can cross or overlap each other. For these reasons, the focus for dense networks has not been placed on identifying single FSs with high spatial precision, but rather on network properties that the FSs form. This change of focus is a result of the challenges in identifying individual FSs in such networks. Different mathematical approaches for tracing of FSs have been formulated for dense networks (Fig. 2). Some of these methods are applicable to networks consisting of FSs characterized by straight to semi-straight rods, while others can also handle FSs that bend.

Here we aim to systematically review algorithms used for identification (*i.e*. tracing) of FSs from segmented image-data. In doing so, we detail the underlying assumptions, along with advantages and limitations, of the different approaches. All statements in this review are based on the original articles published by the developers. Our emphasis also included methods that facilitate the study of dynamic properties of the cytoskeleton. Since algorithms for image segmentation have been covered elsewhere (*e.g.*, Ozdemir et al. [15]), we focus on the algorithms for tracing and tracking of FSs. For systems that do not contain spatially defined single filaments, where FSs are defined based on thickness and branching (*e.g.* roots and vascular tissue), the focus is solely on tracing as these systems are static on a short time-scale.

## 2 Classification of approaches for tracing and tracking of FSs

### 2.1 Approaches applicable to sparse networks

Tracing and tracking of single FSs in sparse networks have been performed using different approaches, ranging from semi- to fully-automated. These approaches have been used to mainly analyze data from *in vitro* experiments involving the cytoskeleton. The cytoskeleton is an intracellular network that consists of microtubule (MT), intermediate and actin filamentous structures (AFSs), which underpin a range of cellular functions [1], [6], [16]. MTs are polymers of tubulins forming a hollow tube, making the structure stiffer than an AFS, which are formed by linear polymers of globular actin [17]. Investigation of single FSs from the cytoskeleton (that are below the diffraction limit of light microscopy) has led to the development of robust approaches to calculate the FS center through deconvolution approaches. Research of how the dynamics of MTs and AFSs change during cell growth and development, with and without the interaction of different associated proteins, is still ongoing [18], [19]. Here, both *in vitro* and *in vivo* studies facilitate understanding of the dynamics of FSs under such different conditions. In this section, we describe different approaches to trace and track FSs in sparse networks.

#### 2.1.1 Semi-automated approaches for FS tracing and tracking

Here we describe the semi-automatic methods for tracing and tracking. In contrast to fully automated approaches, semi-automatic approaches require user interactions.

Smith et al. [20] presented JFilament based on the approach by Li et al. [21], using stretching open active contours (SOACs). SOACs represent parametric curves that are fitted to the intensity peaks forming lines in an image. The tool can trace FSs in both two- and three-dimensional imaging data. However, this method cannot describe network properties nor handle filament crossings. The tool allows both fully and semi-automated FS tracing. The user must initialize a new active contour seed near a filament that will be fitted to the putative FS. The user can decide to approve or manually edit the fitting of the function after the curve fitting.

The method proposed by Demchouk et al. [22] employs line scans to the chosen area of interest, which is then fitted to a Gaussian curve to obtain the positions of MTs per line scan. This method was pioneering in improving the precision of tip tracking for single MTs to 36 nm under standard *in vivo* conditions, achieving a 6-fold improvement to the diffraction limit, and validated with *in silico* data including blur and noise. At the time of this publication, other methods performed at the level of the diffraction limit resolution. This approach requires the user to mark an area that contain the MT tip along with an approximate stop and end position of the MT, which allowed insight into the construction of the MT tip.

Another semi-automatic tool, named FIESTA, was published by Ruhnow et al. [23]. The aim of FIESTA is to obtain nanometer precision for the location of MT center lines as well as tip position for stationary MTs. FIESTA can also be used for tracking of MTs. The algorithm is designed for *in vitro* two-dimensional imaging data, and although the authors infer *in vivo* behaviors, only *in vitro* systems were investigated. Notably, the latest version of FIESTA has 21 parameters that can be set by the user. The method relies on fitting 2D Gaussian distributions to the image intensity to acquire MT positions. FIESTA renders high positional precision at least based on sparse *in vitro* data and can handle crossing FSs. However, to what extent FIESTA can handle FS cross-overs, while still accurately identify them, remains to be seen. Furthermore, it has not been investigated to what extent FS density affects the accuracy of MT identification and tracking.

#### 2.1.2 Automated approaches for FS tracing

Sparse networks have also been investigated with fully automated approaches. Automated methods require less human interaction, which renders less user time to analyze image-data, and introduce less bias in measurements.

In contrast to the semi-automated approaches described above, the approach of Xiao et al. [24] handles an unspecified number of FSs in a fully automated manner. The approach is based on B-spline vector level-sets [25] which provide a mathematical description for the shape of filaments. The approach was developed for sparse FS networks and assumes that the FSs have uniform intensity along their length and that the image is of uniform background intensity. The approach has two user-defined parameters: scale size and regularization. The proposed approach can trace FSs, even when they are bent, with high precision and is robust to noise. A comparative analysis against other semi-automated approaches indicated that this approach performed at least as well in terms of FS tracing.

The fiber finding algorithm (FFA), developed by Rossen et al. [26], can trace single fibrin filaments as well as filaments integrated in larger bundles in sparse as well as dense networks. The signal from the collagen fibers are considered convoluted by the point spread function (PSF) of the optical system. However, correlation with the actual PSF of the optical system can then be used in a deconvolution process. The PSF of light microscope is used as a metric to distinguish the mesh created by the fibrin filaments from background. Finally, each FS is blotted out before the tracing of a new one. The method enhances lines over noise, enabling the analysis of noisy data. Since the focus of this approach was on collagen type 1 fibers, which are static, the approach does not perform FS tracking. Instead, the method investigates fiber mesh systems in 3D where pore size from the mesh of FSs, referring to the empty spaces in the mesh, fiber length and persistence length are properties important for the structure of the mesh system. The approach depends on seven user-defined parameters.

#### 2.1.3 Automated approaches for FS tracing and tracking

Automated approaches for tracing and tracking enables researchers to investigate the temporal dynamics of FSs as well as the spatial positions, aiding in understanding of the system’s dynamics.

Prior to the work of Li et al. [21], dynamic measurements of elongating FSs were typically performed using manual or semi-automated methods. Li et al. implemented an approach using open active contours, mentioned above, to trace and track FSs. This method was intended for data from total internal reflection fluorescence microscopy (TIRFM) that excites fluorescent objects in a thin optical specimen section close to the coverslip at an angle and measures the reflected light. The method is applicable to sparse filament networks, exemplified by polymerizing AFSs *in vitro*. While the method can handle crossing FSs, it remains unclear to what extent FS crossings will be traced correctly. The active contour model was developed by Kass et al. [27], but it was not directly applicable since it links together boundaries of intersecting FSs as it relies on closed curves. By contrast, the approach of Li et al. relies on open rather than closed curves in which a new external energy term was employed, consisting of an image and stretching term that improved the fitting performance compared to the original formulation. The method operates directly on images by employing the spline functions that are fitted to the image intensity.

The automated approach of Kapoor et al. [28], named Mtrack, was developed for TIRFM image-data to measure MT dynamics in *in vitro* experiments. The approach is designed for a specific *in vitro* experimental setup and depends on 11 adjustable parameters of which five are preset. The approach can measure growth and shrinkage of MTs over time from the image chosen as initiation point. It is organized into two modules: the first defines all MT seed positions and track their dynamics and the second interprets the data. The seed detection is performed using maximally stable extremal regions, which is a method of blob detection in images [29], [30]. Tracking is performed using 2D Gaussian polynomial models to determine end points of MT filaments. The MT seeds must be diluted enough to have a resolution of multiple pixels between them, but FSs can cross as they grow over time without compromising their identification.

Moving away from conventional mathematical segmentation and tracing approaches, Masoudi et al. [31] published a method implementing a deep learning approach to trace and track MTs using *in vitro* image-data. The approach is based on recurrent neural network and can be applied in image analysis of MT gliding assay data. The tracking part aims to match points (and thereby filaments) between two time frames, which can be cast as a classical assignment problem [32]. Recurrent neural network, as a supervised machine learning (ML) approach, requires training using ground truth annotated data. However, creating annotated data is a challenging task, and is therefore not often implemented if the spatial structure changes between experiments. While this approach offers an interesting alternative for tracing and tracking of FS in sparse networks, its generalizability has not yet been investigated.

### 2.2 Approaches applicable to dense networks

There are numerous approaches based on different mathematical models to study dense networks of FSs. The focus of these approaches is to understand how the network is structured and how FSs interact with each other over time. Most of the approaches can handle bent FSs; however, a few approaches have used the specific properties of the studied system and focus exclusively on (semi) straight FSs.

#### 2.2.1 Approaches for tracing of semi-straight FS

In comparison to sparse networks, tracing of FSs in dense networks is a more challenging problem. To facilitate tracing, some methods focus solely on tracing of straight filaments, which are easier to identify.

The semi-automated approach called Filament sensor, by Eltzner et al. [33], was designed to extract stress fibers from image-data. Stress fibers appear as semi-straight FSs because of their composition. The approach depends on 32 user-defined parameters and takes as input 2D *in vivo* image-data. It is built on a width-aware segment sensor that, after preprocessing and segmentation, defines width for each foreground pixel. This provides a map of pixels defined to contain the FS, and FSs are identified as the longest straight paths of uniform width.

The approach proposed by Alioscha-Perez et al. [34] was developed to address problems of image related artifacts and heavy blurring in tracing of straight FSs. Based on the assumption that filaments are quasi straight, they implemented the MCALab tool [35] to divide image-data into two sources: texture or noise and cartoon or filaments. After preprocessing and division of image-data, the approach performs edge enhancement on the cartoon part of the image. The proposed algorithm outperforms Filament sensor [33] on which it is loosely based. However, the study does not detail all the settings used to divide image-data into the two specified sources.

#### 2.2.2 Approaches for tracing of bent FSs

The investigation of dense networks *in vivo* to identify FSs has been addressed by conventional, graph theoretical, line- and orientation filter transformations as well as by approaches based on SOACs. In this section we elaborate on these methods grouped according to the mathematical formalism on which they are based.

**Conventional approaches.** The approach termed individual fibre segmentation (IFS) by Qiu et al. [36] was developed to automatically obtain properties of individual FSs from a dense network. IFS preprocesses the image by filament enhancement using the Satos tubeness filter [37], eliminating weak signals and removing short coherent pixel clusters. The approach breaks the filament network into fragments, and then recombines them according to a specific set of rules. The fragmentation and recombination is based on node properties of overlaps with other filaments. This defines individual filaments, even beyond crossing and overlaps.

Fsegment, developed by Rogge et al. [38], is a framework to identify stress fiber bundles in 2D image-data. At the time of their publication, other tools [33], [39] also identified stress fibers. However, they did not capture “bendiness” nor filament width correctly. To resolve these shortcomings, Rogge et al. developed a preprocessing step to enhance FSs, dependent on conventional image filters, followed by tracing of the mask to identify FSs. The tracing step contains a connection filter that allows broken pieces of filaments to be defined as one if the distance and angle is under a certain threshold value. However, no preprocessing step was implemented to deal with noisy input. The approach can manage FS crossing, allowing filaments to be defined as one beyond junction, and smaller overlaps. Filaments that cross with short fragment overlapping are not traced correctly by this approach.

**Graph-based approaches.** The open-source approach NEFI, developed by Dirnberger et al. [40], was designed to extract a mathematical network representation of a FSs (Fig. 2b) from various domains, and thus represents a tool with general applicability. Networks (*i.e*. graphs) provide a mathematical way to represent information of pairwise interacting components. NEFI allows the user to select from a list of algorithms for segmentation and preprocessing. Skeletonization of the images was based on the Guo-Hall approach [41] which thins FSs to one pixel width. In contrast to most other tools that rely on the Zhang-Suen approach [42] which is faster, although less accurate than the Guo-Hall approach. To define a graph representing the FSs from the skeletonized and pre-processed image, NEFI implemented an additional graph filtering step to remove noise. However, the approach does not extract FSs from the graph, does not provide any analyses on the resulting graph, and cannot handle low signal-to-noise data.

DeFiNe, developed by Breuer and Nikoloski [43], was developed to identify FSs in a dense network by decomposing the network into a sets of paths that cover all edges in the network, where the paths represent FSs. DeFiNe requires an undirected weighted graph as input in which tracing of FSs corresponds to solving a path cover problem. A path cover problem aims to identify assignment of each edge to a path, such that the set of so-constructed paths respects constrains related to average intensity and angles on the path. Since finding an optimal path cover is a computationally intractable problem, DeFiNe provides a heuristic solution that relies on sampling paths using a modified breadth-first-search. It then minimizes average roughness of the sampled paths, where roughness is defined as change in edge weight along the paths. The user can allow filaments to overlap or not, without specifying the degree of allowed overlap. DeFiNe minimizes average roughness since this favors shorter paths, assumed to be the best representation to capture relevant FSs. The method depends on seven user-defined parameters.

The automated open-source approach by Breuer et al. [44] and an updated version of the tool by Nowak et al. [45], aims to quantitatively measure the structure of the actin cytoskeleton and how this relates to organelle transport in cells. The proposed method is developed for 2D dense AFSs but can be applied to multiple other FS systems. The method depends on seven user-defined parameters. The approach contains preprocessing steps to enhance FSs, including the Sato filter [37]. The structure is then skeletonized and translated into an undirected weighted graph. The graph is used to calculate morphology and topological properties of the underlying network. The approach allows one to study the relation between dense networks and processes, *e.g.* Golgi transport, taking place on these networks.

To enable investigation of 3D image-data describing protein networks, Asgharzadeh et al. [46] developed a graph-theoretic approach. The 3D image-data is segmented by adaptive local thresholding with user correction which is then translated into a graph representation. Network parameters are then extracted from the graph that contains spatial information. The approach was used to investigate the FtsZ1–2 protein networks in the chloroplast of *Physcomitrella patens*.

Another graph-based approach, FinTA developed by Flormann et al. [47], aimed at tracing AFSs from scanning electron microscopy data as well as data acquired by different light microscopy techniques. The approach depends on eight user-defined parameters and is based on binarization by applying vectorial tracing directly on the image-data, resulting in an increased spatial precision compared to segmentation-based methods. The approach populates the image with a variable number of starting nodes, then calculates second-order derivatives of Gaussian kernels to find the next node location of neighboring filaments. With this approach the entire image is traced out and translated into a graph. This method is designed for ordered FSs that create mesh networks. Thus, the approach allows for quantification of morphological properties of the resulting mesh networks.

**Line-filter and orientation-filter transforms.** Another type of mathematical formulation of FS tracing was proposed by Zhang et al. [48] in the method termed SIFNE. This approach is based on line filter transform (LFT) and orientation filter transform (OFT), building on the work by Sandberg and Brega [49]. The LFT serves to enhance linear features using only the image intensity information. This is followed by application of the OFT, used to explore if neighboring pixels along a maximum angle threshold have similar preferred directions. After the segmentation step each fragment is pieced together based on geometrical constraints of MTs. The method is developed for single-molecule localization microscopy (SMLM) data of dense MT networks, such as TIRFM. However, for successful tracing, the FSs must be visually distinct from each other. The method cannot accurately piece together fragments with high curvature. The user is allowed to manually edit traces in the built-in GUI interface.

Building on SIFNE, Zhang et al. [50] developed a semi-automated method named Stress fiber extraction (SFEX) to investigate stress fibers in cells. After preprocessing and segmentation based on their previous work, the image is skeletonized, all junctions are removed, and the fragments are subsequently reconstructed based on geometrical constraints. This method can define FSs across junctions, dependent on geometry of the underlying structure. The framework allows users to adjust parameter values based on visual inspection. For instance, the user must specify whether thin or thick bundles of FSs are to be investigated, as the width of filaments must be approximately the same. The approach relies on at least 31 parameters and allows quantification of morphological properties of FSs.

Liu et al. [51] created Meshworks Analyzer based on the previous work of SIFNE and SFEX. This tool was developed to analyze the architecture of AFSs in mouse embryonic stem cells, which was performed using SMLM image-data. The tool allows quantification of network properties, and neglects characteristics of individual FSs. The tool is interactive, allowing to modify the parameters. After preprocessing, a noise reduction step is performed on the pores from the mesh of the AFSs. The pores from the mesh is later extracted by a Meyer watershed segmentation [52] for analysis.

**Stretching open active contours (SOACs).** SOAX, developed by Xu et al. [53], is based on the mathematical work from Li et al. [21] of open active contours proposed by Xu et al. [54]. Before the development of SOAX, no tool could study and quantify the geometry and topology of 3D dense networks from *in vivo* image-data. Xu et al. addressed this problem by implementing SOACs, which need multiple initialization seed points for each function. For an explanation of SOACs please refer to the description of the method by Smith et al., given above. The SOACs, representing FSs, follow pixels of high intensity, can go beyond junctions, and may overlap; overlapping SOACs can be merged to trace FSs both in 2D and 3D (Fig. 2c). The approach requires 25 user-defined parameters, allowing tracing of both bent and stiff FSs.

Based on SOAX, Xu et al. [55] proposed TSOAX that allows for tracking of FSs. The tracking step was implemented using an extension of the classical assignment problem that matches FSs extracted in different time frames. More specifically, the tracking of FSs are defined as the path cover which minimizes dissimilarity of all paths. This method requires 26 user defined parameters, allowing for flexibility in network. Both SOAX and TSOAX have no preprocessing step of the image-data to reduce noise or enhance FSs, making the two frameworks sensitive to noisy data.

**Approaches relying on deep learning.** Conventional preprocessing filters to enhance FSs include the Frangi vesselness [12] and Sato [37] filters developed to enhance tube-like structures; another common approach is the multi scale line detection [13] filter. These types of filters are often followed by a binarization and skeletonization step to segment FSs. A different approach to pre-process and segment relies on employing ML techniques. Most ML methods use several parameters that can be tuned in the process of model training, and thus rely on minimal user intervention. However, to ensure good performance a large annotated data set is often needed to train the ML models. The creation of annotated datasets is the main drawback in implementing ML-based approaches to segment image-data.

Investigation of retinal images are important for medical purposes. To address this problem Maninis et al. [56] developed the Deep Retinal Image Understanding (DRIU) method, which provides both retinal vessel and optical disc segmentation to analyze eye data robustly and efficiently. This is achieved by a deep Convolutional Neural Network (CNN) with four layers (Fig. 2d). The authors used the same network architecture for both vessel and optic disc segmentation. To test performance of the model, they compared it to four publicly available retinal annotated image-datasets. For all four datasets the DRIU model outperformed human tracing, and in some cases the so-called false positives found by DRIU turned out to be correct and were missed during human-annotation.

Building on the idea of the DRIU model, Shin et al. [57] developed the vessel graph network (VGN). This model is based on a CNN architecture, adopted from the DRIU model to generate pixelwise features. Two additional components were added, consisting of a graph convolutional network (GCN) for extraction of features of connectivity of neighboring vertices and an inference model to produce the final segmentation. With the addition of a GCN component to the CNN architecture, the method performs at least as good as the DRIU model. This was also shown by comparison to the DRIU method, where the VGN method outperformed DRIU on two retinal image-datasets and an X-ray angiography dataset.

The method by Liu et al. [58] is a deep learning approach aimed for segmenting and identifying individual MTs. To segment the image-data they implemented a modified U-net [59] (Fig. 2e) from previous work [60] and an additional segmentation step was implemented based on a CNN for an orientation-aware instance segmentation. The CNN contains six orientation associated branches, which the authors discovered empirically to be optimal for MTs. Each branch detects pieces of FSs with specific range and orientation, resulting in separation of FSs pieces by spatial orientation. The idea of breaking FSs into pieces and stitching them back together based on structural rules, depending on the biological system, was adopted from SOAX [53] and SIFNE [48], discussed above. All model parameters are determined during model training, rendering the method parameter free, and comparative analysis showed that this method outperformed both SOAX and SIFNE.

Another deep learning based framework is the work by Todorov et al. [61], named Vessel Segmentation & Analysis Pipeline (VesSAP). This method was developed to quantify and analyze brain vasculature 3D confocal image-data using five layers from the CNN architecture. This method requires a full scan of high-resolution 3D brain image-data, obtained by applying the 3DISCO [62] clearing approach. To circumvent the need of large amount of annotated training data, they implemented a transfer learning approach, using synthetic data of vessel-like structures, and then refined the performance on a small number of manually annotated real brain vessel scans. Comparison to human annotation and the Frangi vesselness filter indicated improved performance, with similar results to V-net and U-net architectures.

RootPainter developed by Smith et al. [63] is a modified version of the network architecture from Smith et al. [64], originally designed to segment root networks from rhizotron image-data. The improved method is designed to segment biological data by implementation of a modified U-net (Fig. 2e), with an interactive learning approach. Using an interactive learning approach requires less annotated image-data as the user can correct the model manually until desired results are obtained. The method was tested on three data sets with different shapes, biopores, which are tubular shaped voids in soil, nodules appearing as spherical shapes on root systems, and roots which appear tubular. Segmentation of the three datasets were generated using the interactive learning module and trained from scratch in less than two hours. For each dataset, performance was good compared to human annotation, showing both quality of the model and feasibility of training ML models using sparse annotation with an interactive approach.

Another model developed for the vascular brain tissue in mice was developed by Tahir et al. [65]. This model is specific to 3D two photon emission confocal microscopy (2PM) data, a technique in which a molecule is simultaneously excited by two photons of longer wavelength than what is emitted [66]. The model employs a deep neural network and is based on the V-net architecture, which is similar to a U-net architecture, and incorporates a balanced binary cross entropy loss and total variation regularization on the networks output. 2PM data become noisier and have less signal as one moves further inside a sample. This is circumvented with the algorithm, showing a 3-fold depth improvement compared to state of the art methods for 2PM data of Damseh et al. [67] and Jerman et al. [68].

## 3 Concluding remarks to the classification of approaches

Given our categorization of approaches for tracing and tracking FSs, one question remains: Is there an optimal approach that can be applied in any situation? There is no simple answer to this question, as FSs vary in structure and organization depending on what type of system is investigated. As a result tools tailored to FSs and their network structures are needed to accurately solve FS tracing and tracking problems.

**Fig. 3:**
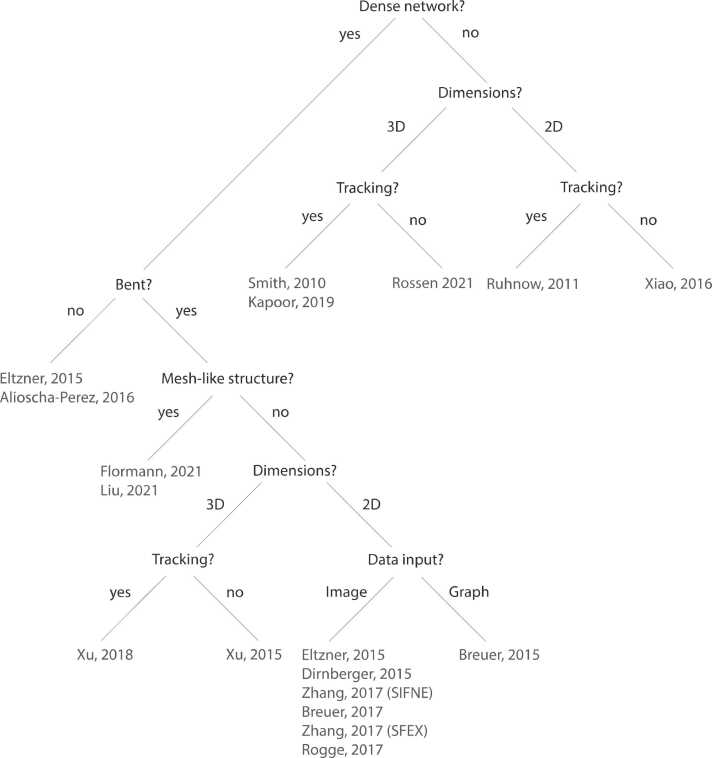
**Decision tree for selecting a suitable framework.** A schematic overview to aid the selection of approaches reviewed in this article. An approach is selected based on the answers in at the internal nodes of the tree.

The evolution of methods for tracing and tracking of FSs in sparse networks has given rise to precise methods (*e.g.*, the Fiji plugin MTrack [28] or FIESTA [23]). However, the main drawback of these tools, with the exception to FFA [26], is that they fail in cases with overlapping FSs in dense networks. Approaches to trace FSs in dense networks either only analyze networks or focus on individual FSs. Preprocessing and segmentation is either followed by a step of piecing together fragments of FSs or direct tracing of FSs. To this end, different methods have been developed depending on whether the aim is to characterize FS network properties or individual FSs. If the investigated system consists of a defined mesh, like collagen type 1 or stress fibers, and network-based characterization is of interest, approaches like FiNTA [47], Meshworks Analyzer [51], or FFA can be used. Since FFA is applicable to both individual and bundled collagen type 1 filaments, one can expect that it can also be used to study AFSs. Yet, graph-theoretic approaches, like NEFI [40] and Cytoseg [44], have proven more suitable for complex networks, typically occurring in the studies of AFS networks. Interested readers can inspect the decision tree provided in Fig. 3 that can be used to select a suitable framework for their problem. While tools based on SOACS, SOAX [53], and the improved version TSOAX [55], can trace and track filaments in dense networks extracted from 3D imaging data, it is unclear how these approaches perform on complex networks from noisy imaging data.

The use of deep learning in image vision is now well-established for segmentation of image-data due to the high precision and lack of user-defined parameters. CNN represents state-of-the-art in image analysis and all deep learning approaches covered in this review have implemented different types of CNN-based models. While these outperform the conventional approaches, they require large annotated datasets. This shortcoming may be overcome by an interactive learning approach (as in RootPainter [63]) to save resources for model training.

## 4 Summary and outlook

From this extensive review of methods covering tracing and tracking for both sparse and dense networks of FS, we identified three open questions that provide fruitful directions for future work; these are related to (1) the mathematical formulation of the network of FSs as well as individual FSs, (2) the development of parameter-free approaches, to reduce bias, and (3) improvement in imaging technology for acquisition of data on FSs.

To the first point, there is currently no consensus on how to mathematically define a network of FSs as well as how to define individual FSs in the network. The latter includes the problems of how to define end and start positions of FSs as well as curvature, and how to handle identification of individual FSs by defining junctions and overlap correctly. As outlined in our review, different techniques have been proposed to tackle these issues, each tailored to a specific system of interest. Several approaches have addressed the issue of defining FSs by defining fragments of a FS and then piecing them together based on structural properties; however, this does not provide a unified model of how FSs can be defined, and then identified, although all existing rules regarding how to stitch pieces together beyond junctions rely on angles and distances. In future work, a framework can be developed inspired by path cover and partition problems that are applicable across different network types.

While a large number of parameter settings renders a method flexible to structurally different systems, it can introduce potential errors in specific applications. This can lead to cases where the same data investigated with a tool with high flexibility by two independent users could lead to highly different results. This issue is currently bypassed by presetting values for the employed parameters, for structural cues such as angles or distances, rendering the method less flexible. Another way to resolve this issue could rely on benchmark data sets that are used for parameter tuning. This could be achieved by considering ML approaches not only for segmentation, but also for tracing of individual FSs. This approach can ultimately combine graph-theoretic and deep learning approaches based on the developments of graph neural networks [69].

Most light microscopy techniques rely on light of a certain wavelength that stimulates a fluorophore or dye in the sample, giving rise to fluorescence and thus emission of light in a specific wavelength being cast back by the sample. The issue with information from light is that the investigated structure usually is smaller than the emitted wavelengths. This challenges the accuracy in determining size and position of an object. This issue has to some extent been dealt with through various deconvolution approaches and *via* super-resolution microscopy. However, such approaches may sometimes introduce image artefacts and are in many cases incompatible with FSs that move. In addition, fluorescence may also be problematic in context of optical blur, bleaching, autofluorescence of samples and signal loss due to depth of sample. Other types of problems occur from the conformational change upon integration of a fluorophore to a protein, causing potential change to the structure, and imaging light sensitive tissues can cause alterations in the sample as it is imaged. Moving to improved light microscopy techniques, like interferometric scattering (iSCAT) [70], [71], or rotating coherent scattering (ROCS) [72], both being label-free microscopy methods, would enable imaging without fluorophores giving rise to fewer problems and faster imaging time. These issues could be dealt with completely by moving on to new technologies that do not use visible light as input, but potentially something else in the future.

Together, these perspectives and systematic reviews of approaches for tracing and tracking FSs indicate that there are several open problems and directions which can stimulate research at the interface of both computational and experimental biology of FSs. Applications of resulting tools can further propel the discovery of functions and properties of FSs in diverse biological systems. (Table 1, Table 2, Table 3).

**Table 1:** **Summary of computational approaches for tracing and tracking FS in sparse networks.** Name refers to the given name of the implemented algorithm, if available. Input indicates the used data structure; if tracking is implemented time-series are used. Network refers to the type of network to which the approach is applicable. Output provides a brief description of the results together with list of outputs if published. Parameter number is the user defined parameters that the approach requires as input; for approaches whose implementation is not publicly available, this number remains unknown. Implementation specifies technical details of the developed tools and the status of their publication. Open source refers to the algorithm being developed in an open source language.

Name	Input	Network	Output	Parameter number	Implementation	Open source	Author and year
	2D time-series	Diluted	Dynamics	Not known	Not published		Li et al., 2009 [21]
			Not known				
JFilament	2D/3D time-series	Diluted	Morphology and dynamics	8	ImageJ	Yes	Smith et al., 2010 [20]
			Coordinates of traces, length, bending, torsional persistence length, and tracked filaments				
	2D time-series	Diluted MT	Dynamics	Not known	Not published		Demchouk et al., 2011 [22]
			Not known				
FIESTA	2D time-series	Diluted MT	MT dynamics	21	Matlab	No	Ruhnow et al., 2011 [23]
			Coordinates of traces, length, and tracked filaments				
	2D image	Diluted	Trace of FS	2	Matlab & Fiji	No	Xiao et al., 2016 [24]
			Traced filaments, and parametric polynomial descriptions of filaments				
	2D time-series	Diluted MT	MT dynamics	Not known	Not published		Masoudi et al., 2019 [31]
			Not known				
MTrack	2D time-series	Diluted	MT dynamics	11	Fiji	Yes	Kapoor et al., 2019 [28]
			Coordinates of traces, length, and tracked filaments				
FFA	3D image	Single & bundles	Collagen type 1 mesh morphology	7	Matlab	No	Rossen et al., 2021 [26]
			Traced filaments, contour length, persistence length, and 3D mesh size

**Table 2:** **Summary of computational approaches for tracing and tracking FSs in dense networks.** Name refers to the given name of the implemented algorithm, if available. Input indicates the used data structure, if tracking is implemented time-series are used. Network refers to the type of network to which the approach is applicable. Output provides a brief description of the results together with list of outputs if published. Parameter number is the user defined parameters that the approach requires as input; for approaches whose implementation is not publicly available, this number remains unknown. Implementation specifies technical details of the developed tools and the status of their publication. Open source refers to the algorithm being developed in an open source language.

Name	Input	Network	Output	Parameter number	Implementation	Open source	Author and year
IFS	2D	Bundles	Morphology	Not known	Not published		Qiu et al., 2014 [36]
			Not known				
Filament Sensor	2D	Bundles	Morphology	32	Java	Yes	Eltzner et al., 2015 [33]
			Coordinates of traces, orientation, length, and width				
DeFiNe	Weighted graph	NA	Weighted graph with trace of FS	7	Python 2.7 (source code updated to 3)	Yes	Breuer and Nikoloski, 2015 [43]
			Set of paths defining traced filaments				
NEFI	2D	Bundles	Weighted graph	NA	Python 3.4	Yes	Dirnberger et al., 2015 [40]
			Weighted undirected planar graph populated with edge length and edge widths				
SOAX	2D & 3D	Bundles	Morphology and trace	25	GUI	Yes	Xu et al., 2015 [53]
			Coordinates of traces, network curvature, length, point density, radial orientation, and spherical orientation				
	2D	Bundles	Morphology	Not known	Not published		Alioscha-Perez et al., 2016 [34]
			Not known				
SIFNE	2D SMLM	Bundles	Trace of FS	NA	Matlab with GUI	No	Zhang et al., 2017 [48]
			coordinates of traces, junctions, curvature, and length				
Cytoseg	2D	Bundles	Morphology of network	7	Python 3 & Fiji	Yes	Breuer et al., 2017 [44]
			Populated weighted undirected graph and information of nodes, edges, connected components, avg. edge capacity, assortativity, avg. path length, CV path length, algebraic connectivity, CV edge angle and crossing number				
SFEX	2D	Bundles	Morphology and trace of FS	31	Matlab	No	Zhang et al., 2017 [50]
			Coordinates of traces, orientation, length, centroid and width				
Fsegment	2D	Bundles	Morphology and trace of FS	2	Matlab	No	Rogge et al., 2017 [38]
			Coordinates of traces, length, width, orientation, and intensity				
	3D	Bundles	Morphology and graph parameters	Not known	Not published		Asgharzadeh et al., 2018 [46]
			Not known				
TSOAX	2D & 3D timeseries	Bundles	Trace and intensity of FS	26	GUI	Yes	Xu et al., 2019 [55]
			Coordinates of traces, pixel values, and tracked filaments				
FiNTA	2D	Bundles	Mesh and filament morphology	8	C+ +	Yes	Flormann et al., 2021 [47]
			Mesh hole size, circularity of mesh hole, junction distance, density, length, connectivity of each unified junction, global angle distribution, and persistence length				
Meshworks Analyzer	2D	Bundles	Mesh morphology	NA	Matlab & IDL	No	Liu et al., 2021 [51]
			Pore area, pore edge length, pore vertex angle, and filament density

**Table 3:** **Summary of deep learning approaches for tracing FS in dense networks.** Name refers to the given name of the implemented algorithm, if available. Input indicates the used data structure. Approach states what machine learning architecture that was implemented. Implementation specifies technical details of the developed tools and the status of their publication. Open source refers to the algorithm being developed in an open source language.

Name	Input	Approach	Implementation	Open source	Author and year
DRIU	2D retinal data	CNN	Python 2	Yes	Maninis et al., 2016 [56]
VGN	2D vessel-like data	CNN, GCN	Python 2	Yes	Shin et al., 2019 [57]
Not named	2D MT data	U-net, CNN	Python 3, Matlab	No	Liu et al., 2019 [58]
VesSAP	3D brain vasculature data	CNN	Python 3, Matlab	No	Todorov et al., 2020 [61]
RootPainter	2D biological data	U-Net	GUI, python 3	Yes	Smith et al., 2020 [63]
Not named	3D 2PM brain vasculature data	V-net	Python 3	Yes	Tahir et al., 2021 [65]

## CRediT authorship contribution statement

Conceptualization: Z.N. Formal analysis: I.O., Z.N. Funding acquisition: S.P. Methodology: I.O. Supervision: S.P., Z.N.; Visualization: I.O. Roles/Writing - original draft: I.O., Z.N.; Writing - review & editing: all authors.

## Conflict of interest

The authors declare no conflict of interest.
